# Seasonal Dynamics of Ticks and Tick-Borne Pathogens in Republic of Korea

**DOI:** 10.3390/pathogens13121079

**Published:** 2024-12-08

**Authors:** Sezim Monoldorova, Sungkyeong Lee, Seungri Yun, Sunho Park, Jong-Uk Jeong, Jiro Kim, In-Yong Lee, Hojong Jun, Chan-Ho Park, Hyeon-Seop Byeon, Mina Han, So-Youn Youn, Yun-Sang Cho, Young-Min Yun, Kwang-Jun Lee, Bo-Young Jeon

**Affiliations:** 1 Department of Biomedical Laboratory Science, College of Software and Digital Healthcare Convergence, Yonsei University, Wonju 26493, Republic of Korea; 2 Department of Clinical Laboratory Science, Cheju Halla University, Jeju 63092, Republic of Korea; 3 Department of Tropical Medicine and Institute of Tropical Medicine, Yonsei University College of Medicine, Seoul 03722, Republic of Korea; 4 Department of Medical Environmental Biology and Tropical Medicine, School of Medicine, Kangwon National University, Chuncheon 24341, Republic of Korea; 5 Gangwon State Veterinary Service & Research Institute, Chuncheon 24203, Republic of Korea; 6 Chungbuk Province Veterinary Service & Research Institute, Cheongju 28135, Republic of Korea; 7 Bacterial and Parasitic Disease Division, Department of Animal & Plant Health Research, Animal and Plant Quarantine Agency, Gimcheon 39660, Republic of Korea; 8 Department of Veterinary Internal Medicine, College of Veterinary Medicine, Jeju National University, Jeju 63243, Republic of Korea; 9 Division of Zoonotic and Vector Borne Disease Research, Center for Infectious Diseases Research, Korea National Institute of Health, Cheongju 28159, Republic of Korea

**Keywords:** ticks, *Haemaphysalis longicornis*, *Borrelia*, *Anaplasma*, *Theileria*

## Abstract

Tick-borne diseases are a public health problem and a significant burden on the livestock industry. The seasonal abundance of ticks and tick-borne pathogens strongly correlates with the prevalence of these diseases. To investigate the seasonal variation in ticks and tick-borne pathogens, ticks were collected from Gangwon State, Korea, and the tick-borne pathogens *Borrelia*, *Anaplasma*, *Babesia*, and *Theileria* were examined. In total, 14,748 ticks were collected, comprising ticks from two genera and three species: *Haemaphysalis longicornis*, *Haemaphysalis flava*, and *Ixodes nipponensis*, with *H*. *longicornis* being the predominant species. Of 7445 ticks (455 pools) examined for pathogens, *Theileria* was detected in 61 pools, whereas *Borrelia* and *Anaplasma* were observed in 17 pools. *H*. *longicornis* nymphs and adults were collected beginning in April, with nymph numbers peaking in May and June and adult ticks peaking in June and July. In contrast, the larvae were collected in May and peaked in September. Tick-borne pathogens were detected in April, peaking in July and September. *Borrelia*, the causative agent of Lyme disease, exhibits a temporal association between its detection in ticks and its occurrence in humans. In conclusion, tick-borne diseases seem to be closely linked not only to changes in tick numbers throughout the seasons but also to the seasonal variations of the pathogens within them.

## 1. Introduction

Ticks are obligate blood-sucking arthropods that feed on the blood of mammals, birds, reptiles, and amphibians and can transmit viruses, bacteria, and protozoa [1]. Ticks are important vectors for various diseases that affect humans, livestock, and other animals. The risk of tick-borne disease transmission is increasing worldwide, and tick-borne pathogens are becoming increasingly important as they cause zoonotic diseases that affect humans, livestock, and wildlife [2].

Ticks transmit bacterial diseases such as ehrlichiosis, bartonellosis, Q fever, Lyme disease, anaplasmosis, and tick-borne rickettsiosis; protozoal diseases including babesi- osis and theileriosis; and viral diseases such as Crimean-Congo hemorrhagic fever, severe fever with thrombocytopenia (SFTS), tick-borne encephalitis, Heartland, and Powassan virus disease [1,2,3].

*Borrelia* is a tick-borne spirochete that causes Lyme disease, clinically expressed by fever, erythema migrans, musculoskeletal pain, and neurological symptoms in humans [4]. *Borrelia* spp. are divided into the Lyme disease group, which includes *Borrelia burgdorferi* sensu lato, and the relapsing fever group, with the reptile group recently added [5]. The Korea Disease Control and Prevention Agency designated Lyme disease a statutory infectious disease in 2010 and it occurs continually yearly [6]. *B*. *burgdorferi*, a causative agent of Lyme disease, was first isolated from *Ixodes* ticks in Korea in 1992 [7], and *B*. *burgdorferi* sensu stricto, *Borrelia afzelii*, and *Borrelia garinii* were detected in ticks of *Ixodes nippponensis*, *Haemaphysalis longicornis*, and *Amblyoma* [8].

*Anaplasma* is a Gram-negative intracellular bacteria that causes an acute febrile illness known as anaplasmosis in animals or human granulocytic anaplasmosis [9]. Human granulocytic anaplasmosis was reported in the United States in 1994 and in 2014 in Korea, where it occurs sporadically [10,11,12]. In animals, *A*. *phagocytophilum* infection has been reported in dogs [13], and *A*. *bovis* and *A*. *carpa* infections have been reported in cattle and goats [14]. *I*. *scapularis* and *I*. *ricinus* are vectors of *Anaplasma* in the United States [15] and western Europe [16], and *Anaplasma* has been detected in *H*. *longicornis*, *I*. *nipponensis*, and *I*. *persulcatus* in Korea [17].

*Babesia* and *Theileria* are intraerythrocytic piroplasmas that belong to the phylum Apicomplexa. *Babesia* is a zoonotic pathogen that causes babesiosis in humans and animals, whereas *Theileria* causes infections in livestock and wild animals. In humans, *Babesia microti*, *Babesia divergens*, and *Babesia venatorum* cause infections [18], whereas *Babesia canis* and *Babesia gibsonii* infect dogs [19]. In Korea, a case of human babesiosis was reported in 2005 [20]. *Theileria annulata* and *Theileria parva* cause tropical theileriosis and East Coast fever in cattle, respectively [21]. Additionally, *Theileria* species, such as *T*. *orientalis*, *T*. *luwenshuni*, and *T*. *ovis* cause anemia, jaundice, and anorexia in domestic animals and wildlife [22]. The main vectors of *Babesia* and *Theileria* are *Ixodes ricinus* and *Ixodes scapularis* in Europe and the United States, respectively [23].

Tick-borne diseases are influenced by a variety of factors, including ticks and their life cycle, pathogens, hosts, and climate. In tropical and subtropical regions, ticks live year-round; however, in temperate regions, tick life cycles are seasonal, which may be related to the occurrence of tick-borne diseases. Although many studies have been conducted on the detection of pathogens in ticks, very few have focused on seasonal changes in ticks and pathogens and their relevance to outbreaks of tick-borne diseases.

This study aimed to investigate the seasonal prevalence of ticks and tick-borne pathogens, namely *Borrelia*, *Anaplasma*, *Babesia*, and *Theileria,* in Korea and their association with human or animal outbreaks.

## 2. Results

### 2.1. Species of Ticks and Their Seasonal Prevalence

**Figure 1:**
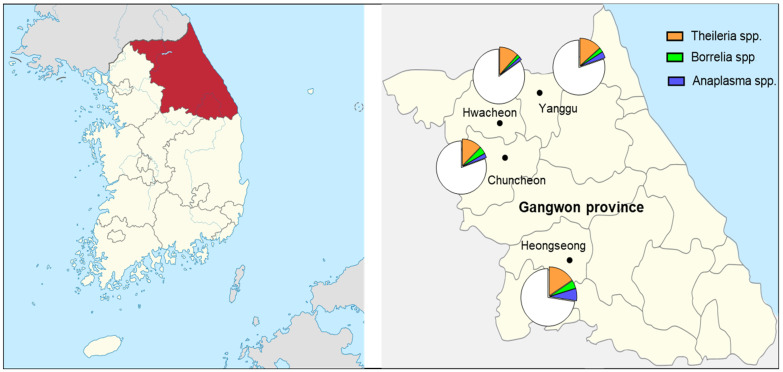
Map of tick collection sites and geographical distribution of tick-borne pathogens in ticks collected in Gangwon Sate, Korea (areas highlighted in red). Collected ticks were pooled, each pool consisting of 1–5 adult ticks, 1–30 nymphs, or 1–50 larvae, and tested for tick-borne pathogens *Borrelia* spp., *Anaplasma* spp., and *Theileria* spp. using PCR.

A total of 14,784 ticks were collected from four localities in Gangwon State, Korea (Figure 1 and Table 1). The collected ticks belonged to two genera and three species, *H*. *longicornis*, *H*. *flava*, and *I*. *nipponensis*. Overall, *H*. *longicornis* (adults 1130, nymphs 11,596, larvae 1868) accounted for 98.7%, followed by *H*. *flava* (1.1%; adults 13, nymphs 154), and *I*. *nipponensis* (0.2%; adults 21, nymphs 2).

The highest number of ticks was collected in Chuncheon, accounting for 58.2% (8554 ticks), followed by Hoengseong with 2995 ticks (20.3%), Yanggu with 2400 ticks (16.5%), and Hwacheon with 750 ticks (5.1%).

**Figure 2:**
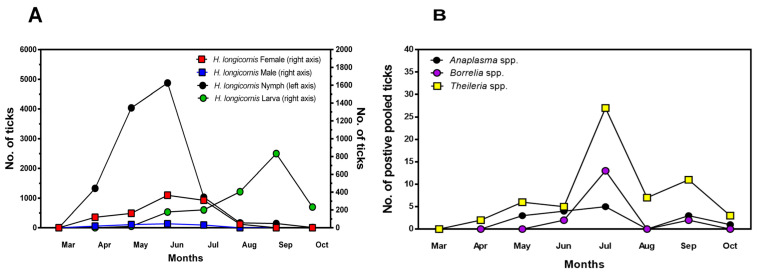
Seasonal distribution of ticks (**A**) and tick-borne pathogens (**B**) in ticks collected in Gangwon State, Korea. Collected ticks were pooled and tested for tick-borne pathogens *Borrelia* spp., *Anaplasma* spp., and *Theileria* spp. using PCR.

The seasonal distributions of *H*. *longicornis*, *H*. *flava*, and *I*. *niponensis* are presented in Table 2 and Figure 2. Adult female and male *H*. *longicornis* ticks were collected from April to July and from April to September, respectively, with more frequent collection from April to July. Nymphs were collected from April to October, with a high number collected from April to July, with peaks in May and June. Larvae were collected from May to October, reaching a peak in September. *H*. *flava* nymphs were collected from April to October, with a peak in May. A few *I. nipponensis* samples were also collected.

### 2.2. Detection of Tick-Borne Pathogens

A total of 455 pools from 7445 ticks were tested for tick-borne pathogens, *Borrelia* spp., *Anaplasma* spp., and *Babesia*/*Theileria* spp. (Table 3). Of the 455 tick pools tested, 17 (infection rate [IR], 3.74%; minimum infection rate [MIR], 0.23%) and 16 (IR, 3.52%; MIR, 0.21%) were positive for *Borrelia* spp. and *Anaplasma* spp., respectively. *Theileria* spp. were detected most frequently, with 61 positive pools of the 455 tick pools (IR: 13.41%, MIR:0.82%) (*Theileria* spp. vs *Borrelia* spp.: χ2 = 27.2, *p* < 0.001; *Theileria* spp. vs *Anaplasma* spp.: χ2 = 28.7, *p* < 0.001).

Both *Borrelia* spp. and *Anaplasma* spp. were primarily detected from *H*. *longicornis*; *H*. *flava* and *I*. *niponensis* were also positive for *Borrelia* spp. and *Anaplasma* spp. *Borrelia* spp. was detected in all developmental stages of *H*. *longicornis*, whereas *Anaplasma* spp. were detected in all developmental stages except in male adult ticks. *Anaplasma* spp. were identified as *A*. *phagocytophilum* through sequencing; however, the *Borrelia* spp.-positive PCR products could not be sequenced.

Of the 61 *Theileria* spp.-positive pools, 58 belonged to *H*. *longicornis*, accounting for 95.1%, and 1 (1.6%) and 2 pools (3.3%) belonged to *H*. *flava* and *I*. *niponensis*, respectively (*H*. *longicornis* vs. *H*. *flava*: χ2 = 4.6, *p* < 0.05). Of the 188 pools of female adult *H*. *longicornis* ticks, 20 (IR: 16.95%, MIR: 3.93%) were positive for *Theileria* spp., and 2 of 28 pools (IR: 7.14%, MIR: 2.82%) of male adult ticks were *Theileria* spp.-positive. In *H*. *longicornis* nymphs, 28 of 222 pools (IR: 7.14%, MIR: 2.82%) were *Theileria* spp.-positive and 8 of 35 pools (IR: 22.86%, MIR: 0.85%) of *H*. *longicornis* larvae were *Theileria* spp.-positive. *Theileria* spp. were detected in all developmental stages of *H*. *longicornis*, with the highest positive IR, 22.86% in *H*. *longicornis* larvae; there was no significant difference in *Theileria*-positive rates among tick developmenthal stages. Sixteen *Theileria* spp.-positive PCR products were successfully sequenced, 12 of which were identified as *T. luwenshuni* and 4 as *T*. *capreoli*. *Theileria* spp. IRs were high in ticks, but *Babesia* spp. were not detected.

In the regional distribution of tick-borne pathogens, the IR of *Theileria* spp. by region ranged from 12.07% to 15.91% (Table 3 and Figure 1). By region, the IRs of ticks for *Borrelia* spp. and *Anaplasma* spp. ranged from 1.72% to 4.55% and 1.72% to 6.82%, respectively. In Hoengseong, where the cattle pastures were located, the infection rates of *Theileria* spp., *Borrelia* spp., and *Anaplasma* spp. were somewhat high, but there were no significant differences compared to other localities.

### 2.3. Seasonal Distribution of Ticks and Tick-Borne Pathogens

The seasonal distribution of tick-borne pathogens, *Borrelia* spp., *Anaplasma* spp., and *Theileria* spp. are shown in Figure 2B. *Borrelia* spp. were detected in June and peaked in July. However, there was a slight increase in Borrelia-positive ticks in September, with no ticks testing positive in August. *Anaplasma* spp. were detected from May to October except August. *Theileria* spp. was detected in ticks from April to October, peaking in July, decreasing dramatically in August, and increasing again in September. Tick-borne pathogens *Borrelia* spp., *Anaplasma* spp., and *Theileria* spp. showed similar patterns, peaking in July, decreasing or not being detected in August, and showing a slight increase in September.

**Figure 3:**
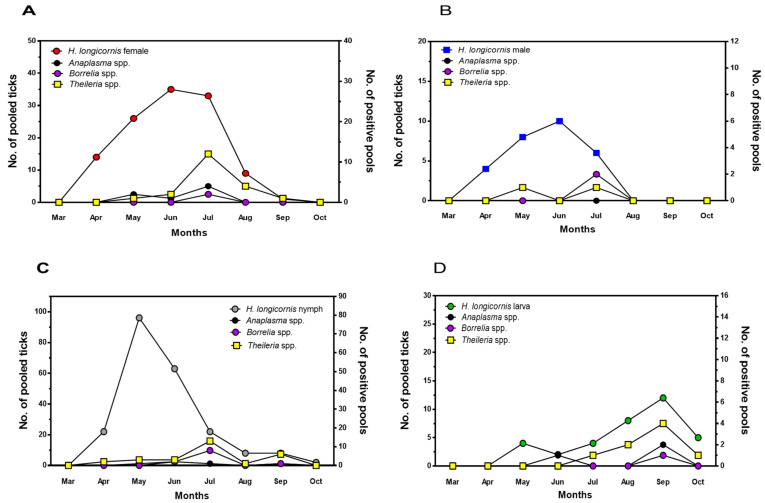
Seasonal distribution of tick-borne pathogens according to the developmental stage of ticks collected in Gangwon State, Korea. Collected female adults (**A**), male adults (**B**), nymphs (**C**), and larvae (**D**) were pooled according to the developmental stage of the ticks and tested for tick-borne pathogens *Borrelia* spp., *Anaplasma* spp., and *Theileria* spp. using PCR.

The seasonal changes in tick-borne pathogens during the developmental stages of *H*. *longicornis*, the predominant tick species, are shown in Figure 3. The number of *H*. *longicornis* adult females and males increased rapidly from March and peaked in June, whereas tick-borne pathogens were detected in May, peaked in July, and then decreased. The number of nymphs peaked in May and showed a sharp decline, and tick-borne pathogens were detected in July and September, when the number of nymphs decreased. Larvae were detected from May and peaked in September. For larvae, the detection of tick-borne pathogens was proportional to the number of larvae, but for adults and nymphs, it was not proportional to the number of ticks and increased at a certain point in time, namely in July.

**Figure 4:**
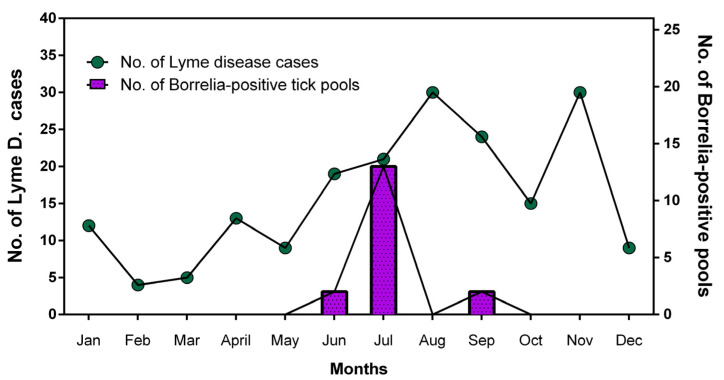
Seasonal distribution of human cases of Lyme disease in Korea from 2011 to 2024 and number of *Borrelia* spp.-positive ticks collected in Gangwon state, Korea. Human Lyme disease cases in Korea were based on Statistical Information on Outbreaks of Infectious Disease, KDCA [6] and *Borrelia* spp. were detected from ticks collected in Gangwon state, Korea using PCR.

To examine the relationship between tick-borne *Borrelia* spp. and human Lyme disease, we compared their seasonal patterns (Figure 4). *Borrelia* spp. were detected in ticks in June, peaked in July, and increased again in September. In the Lyme disease data reported to the Korea Disease Control and Prevention Agency, Lyme disease increased from March, peaked in August, decreased in October, and then increased again in November. The detection of *Borrelia* spp. in ticks showed peaks in July and September, whereas human Lyme disease had peaks in August and November, with an interval of approximately a month. This might be related to the incubation period of Lyme disease, which is the period from *Borrelia* spp. infection via a tick biting to the onset of symptoms of human borreliosis.

### 2.4. Phylogenetic Analysis of Tick-Borne Pathogens

**Figure 5:**
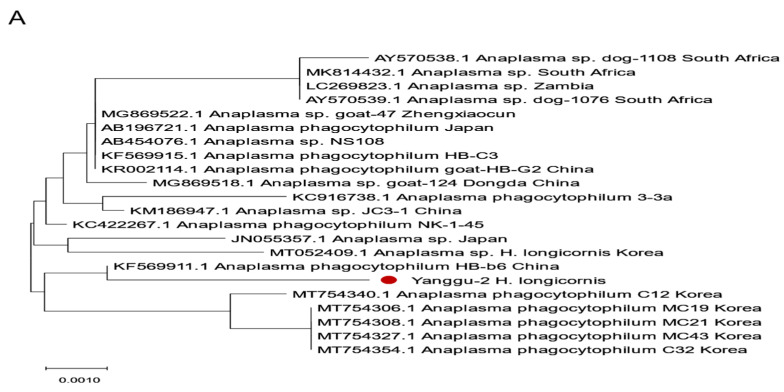

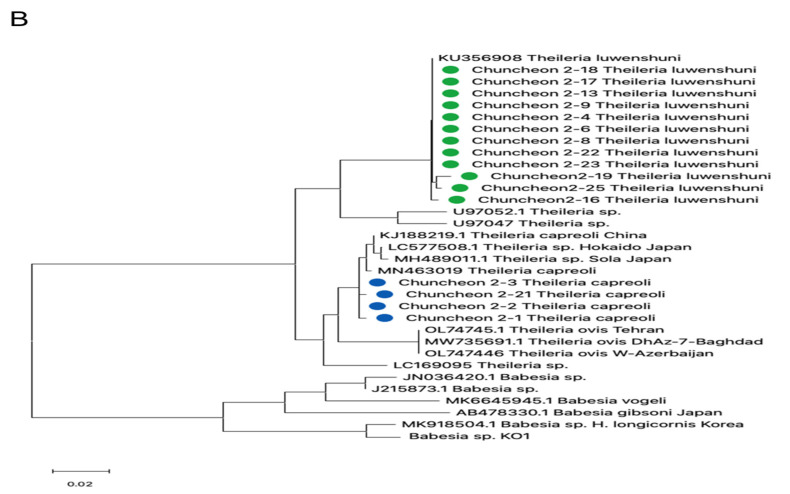
Phylogenetic analysis of *Anaplasma* spp. (**A**) and *Theileria* spp. sequences (**B**) from ticks in Gangwon State, Korea. The phylogenetic trees were generated by MEGA-X (ver. 7.0) software using the maximum likelihood (ML) method. The reliability of the ML trees was assessed by bootstrap analysis with 1000 replicates based on partial sequences of 16S rRNA for *Anaplasma* spp. and 18S rRNA for *Theileria* spp. The sequences of tick-borne pathogens from ticks analyzed in this study are indicated by circles (red circle; *Anaplasma phagocytophilum*, green circle; *Theileria luwenshuni*, blue circle; *Theileria capreoli*).

Phylogenetic analysis of *Anaplasma* spp. and *Theileria* spp. was performed based on the sequences of 16S rRNA and 18S rRNA, respectively (Figure 5). *Theileria* spp. detected in *H*. *longicornis* ticks were divided into *Theileria luwenshuni* and *Theileria capreoli* (Figure 5A). *Theileria luwenshuni* sequences were highly homologous to a *Theileria luwenshuni* sequence (KU356908) detected from deer keds in Korea with an identity value of 98.9–99.5%, and *Theileria capreoli* sequences were close to *Theileria capreoli* sequences, MN463019 from red deer in Turkey and KJ188219.1 from Qilian Mountain red deer in China, with identity values of 95.6–96.0% and 95.8–96.1%, respectively, and appeared to form a new cluster.

*Anaplasma* spp. detected from the ticks in Yaggu were identified as A. *phagocytophilum*, which is very close to KF569911.1 found in China and also close to *A*. *phagocytophilum* sequences reported in Korea.

## 3. Discussion

In this study, we investigated the bacterial and protozoan tick-borne pathogens, *Borrelia* spp., *Anaplasma* spp., and *Babesia*/*Theileria* spp., from ticks collected in Gangwon province, a forested area in Korea.

Among the ticks collected in this study, *H*. *longicornis* was predominant, followed by *H*. *flava*, and *I*. *niponensis*. At the Chuncheon sites, the number of collected ticks was highest, but there was no significant difference in the numbers of collected ticks between collection sites located near cattle and goat farms. This is consistent with several previous reports that *H*. *longicornis* is a major tick in Korea [17]; the high tick density in Chuncheon is likely related to the fact that SFTS, a tick-borne disease, occurred in this region with the highest number of cases in Gangwon province [24].

Ticks collected were tested for *Borrelia* spp., *Anaplasma* spp., and *Babesia*/*Theileria* spp. using PCR, and *Borrelia* spp., *Anaplasma* spp., and *Theileria* spp. were detected, but not *Babesia* spp. *Borrelia* spp., the causative agents of Lyme disease, were detected in *H*. *longicornis* and *H*. *flava*, and the infection rate was 3.97% (MIR, 0.22%) and 3.13% (MIR, 1.06%), respectively. *Borrelia* spp. was detected in all collection sites in Gangwon State and is presumed to be widespread. This is consistent with the fact that *Borrelia afzeli* was detected in *I*. *nipponensis* and *H*. *longicornis* [25,26]. In particular, infection rate of *Borrelia* in *I*. *nipponensis* (MIR: 0.34%) was high [27]. This could be evidence that the first case of Lyme disease was reported in Gangwon State, and it occurs every year [6].

*Anaplasma* spp. were detected in *H*. *longicornis* and *I*. *nipponensis*, with infection rates of 3.72% (MIR, 0.20%) and 5% (MIR, 5%), respectively, but not in *H*. *flava*. *I*. *scapularis* and *I*. *ricinus* have been reported to be the vectors of *Anaplasma* spp. [28]. In Korea, *Anaplasma phagocytophilum* has been detected in *H*. *longicornis*, *I*. *nipponensis*, and *I*. *persulcatus* [29]. Because *H*. *longicornis* is the predominant tick in Korea, it is considered the primary vector of *Anaplasma* spp. and may transmit *Anaplasma* spp. not only in humans but also in dogs, cattle, and goats.

*Theileria* spp. were detected in *H*. *longicornis*, *H*. *flava*, and *I*. *niponensis*, and the average infection rate of *Theileria* spp. in ticks was quite high at 13.41% (MIR: 0.82%) (Table 2). *Theileria* spp. were detected at all collected sites and are presumed to be widely distributed throughout Gangwon State. Two *Theileria* spp. were detected in the sequence analysis: *T. luwenshuni* and *T. capreoli*. In Korea, *T. orientalis* has been detected in cattle and *T*. *luwenshuni* has been detected in cattle and deer [30,31,32]. In the previous study in Korea, *Theileria* was detected in *H*. *longicornis*, *H*. *flava*, and *I*. *nipponesis*, and the infection rate of *Theileria* in *H*. *longicornis* was 39% (MIR: 3.05%) [33]. This suggests that *T*. *luwenshuni* is transmitted from ticks to domestic cattle, wild animals, and deer. In contrast, *T*. *capreoli* was detected in the present study, which is presumed to be the first report of its kind in Korea.

The tick-borne pathogens *Borrelia* spp., *Anaplasma* spp., and *Theileria* spp. demonstrated dynamic seasonal changes. Tick-borne pathogens were associated with the tick life cycle but were not proportional to the number of ticks. *H*. *longicornis* adults and nymphs peaked in May–June, but pathogen detection peaked later in July. Notably, larval and pathogen detection peaked simultaneously in September and showed a close relationship. This suggests that there is a slight temporal delay between tick populations and pathogens in *H*. *longicornis* adults and nymphs, whereas there seems to be a consistent pattern in the case of larvae. There are a couple of reports on the association between ticks and tick-borne pathogens. Seo et al. and Jang et al. showed that the number of ticks and the possession of pathogens are not proportional [34,35]. Seo et al. analyzed the prevalence of ticks and positive rates of SFTSV in ticks [34]. Interestingly, the number of collected ticks increased from April, but the detection rate of SFTSV in ticks decreased inversely. In addition, the number of *H*. *longicornis* larvae increased significantly in September, but the detection rates of SFTSV in larvae decreased sharply. Jang et al. collected ticks from wild animals and detected SFTSV in ticks. The number of ticks collected from wild animals increased significantly in June and September, whereas the SFTSV in ticks was detected in May and September. This indicates that the prevalence of ticks and the detection of pathogen are not proportional. This implies that ticks do not always carry pathogens and might get pathogens by sucking blood from wild animals [36]. Further research is needed on the transmission of tick-borne pathogens between wildlife and ticks.

Lyme disease was designated as a statutory infectious disease in 2010 in Korea, and outbreaks have continued since the first case in 2012. The seasonal incidence of Lyme disease increased in March, peaked in August, decreased in October, increased again in November, and then decreased thereafter (Figure 4). Among ticks, *Borrelia* spp. were detected in June, peaked in July, declined in August, and increased again in September. There seems to be a gap of approximately a month between the peak period of *Borrelia* spp. in ticks in July and the peak period of Lyme disease in humans in August. This may be related to the incubation period after the *Borrelia* spp. infection.

In Korea, there have been a few reports of *Anaplasma* spp. infection in humans and dogs with tick bites [12,13]. In contrast, *Theileria* spp. have been detected in ticks, but infection is rarely reported in livestock, such as cattle and goats. This phenomenon may be related to the change from grazing to housing in barns.

The populations of the tick-borne pathogens *Borrelia* spp., *Anaplasma* spp., and *Theileria* spp. increased until July, peaked, decreased in August, and then increased again. This might be related to the summer rainy season. Korea belongs to a temperate zone, and there is a long rainy season in summer and temperatures drop below zero in winter. The long rainy season lasts from mid-July to early or mid-August. Excessive rainfall or long-term rain during this period seems to reduce tick populations. The number of ticks and pathogens peaks in July, before the rainy season, and then decreases during the long rainy season. Then, the number of larvae increases and reaches a peak in September. As the weather gets colder, the number of larvae decreases rapidly.

In conclusion, ticks were distributed throughout Gangwon State and the main species was *H. longicornis*. Tick-borne pathogens *Borrelia* spp., *Anaplama* spp., and *Theileria* spp. were detected, and the seasonal pattern of tick-borne pathogens, particularly *Borrelia* spp., appeared to be associated with the outbreak pattern of human Lyme disease. This provides evidence of a close link between tick-borne pathogens and infections caused by these pathogens in humans and animals.

## 4. Materials and Methods

### 4.1. Surveillance Localities and Period

Ticks were collected at four locations: Hoengseong (37°29′41″, 128°10′09″), Yanggu (38°13′32″, 128°04′27″), Hwacheon (38°04′07″, 127°47′47″), and Chuncheon (37°56′14″, 127°46′54″) in Gangwon State, Republic of Korea, to conduct tick-borne disease surveillance from March to October 2022 (Figure 1). The collection sites in Hoengseong, Chuncheon, and Hwacheon were near cattle farms, whereas those in Yanggu were near goat farms.

### 4.2. Tick Collection and Species Identification

Ticks were collected using carbon dioxide (CO_2_) gas-based tick traps (Shin-Young Commerce System, Namyangju, Republic of Korea) with dry ice. The traps were made of white tarpaulin and were cylindrical in shape, 36 cm in diameter, and 50 cm in height with an open top. A cylindrical cooler, 25 cm in diameter and 30 cm in height, containing approximately 2 kg of dry ice was placed inside the trap, and CO_2_ was released to attract the ticks. Three traps were set up at each collection site; the traps were placed between 12:00 and 16:00, and the attracted ticks were subsequently collected.

The collected ticks were examined under a stereomicroscope and identified using morphological criteria for the species and developmental stages, according to the taxonomic key [37].

### 4.3. Tick Homogenization and DNA Extraction

The ticks were pooled according to species and developmental stage for pathogen detection. The numbers of pooled ticks were 1–50 larvae, 1–30 nymphs, and 1–5 adults (male/female).

The pooled ticks were homogenized with Zirconia 3 mm beads with 200 μL lysis buffer (iNtRON Biotechnology, Seongnam, Republic of Korea) using a Precellys Evolution Touch Homogenizer (Bertin Technologies, Montigny-le-Bretonneux, France) with two cycles of 20 s at 6500 rpm. Following homogenization, the tubes were centrifuged at 16,200× *g* for 1 min, and DNA was extracted using a G-spin Total DNA extraction kit (iNtRON Biotechnology, Seongnam, Korea) according to the manufacturer’s instructions and stored at –70 °C until further analyses.

### 4.4. Detection of Tick-Borne Pathogens by Polymerase Chain Reaction

The detection of tick-borne pathogens was performed using polymerase chain reaction (PCR) or nested PCR for *Borrelia* spp., *Anaplasma*, and *Babesia/Theileria* spp. using a T100 Thermal Cycler (Bio-Rad Laboratories, Hercules, CA, USA).

To detect *Borrelia* spp. in ticks, nested PCR with two sets of primers targeting *flagellin* B and the 5S-23S *rrf*-*rrl* intergenic spacer region was performed (Table 4) [38,39].

PCR was performed using a AccuPower HotStart PCR Premix (Bioneer Co., Daejeon, Republic of Korea). Each PCR reaction was carried out in a 20 μL reaction volume containing 1 µL of 10 nM of each primer, 13 µL of nuclease-free water, and 5 µL of template DNA in a freeze-dried PCR Premix (Bioneer Co.). The amplification conditions for nested PCR were as follows: The first reaction was performed as an initial denaturation at 95 °C for 3 min, followed by 30 cycles of 30 s at 94 °C, 45 s at 50 °C, and 45 s at 72 °C. This was followed by the second reaction: 3 min at 95 °C followed by 35 cycles of 30 s at 95 °C, 30 s at 54 °C, and 45 min at 72 °C, with a final extension at 72 °C for 10 min.

*Anaplasma* spp. was detected using nested PCR with two sets of primers targeting a fragment of the 16S rRNA gene, as described by Barlough et al. [40] (Table 4). Amplification was performed as follows: an initial denaturation at 95 °C for 3 min, followed by 30 cycles of 30 s at 94 °C, 30 s at 54 °C, and 1 min at 72 °C. This was followed by a second reaction: 3 min at 95 °C, 30 cycles of 30 s at 95 °C, 30 s at 57 °C, and 1 min at 72 °C, with a final extension at 72 °C for 10 min.

To detect the *Babesia*/*Theileria* spp. in ticks, PCR was performed using primers targeting the V4 hypervariable region of 18S rRNA [41]. The thermal conditions for *Babesia*/*Theileria* spp. were as follows: initial denaturation at 94 °C for 3 min; 35 cycles of 1 min at 94 °C, 1 min at 55 °C, and 2 min at 72 °C; and a final extension at 72 °C for 10 min. PCR products were analyzed using gel electrophoresis on 1.5% agarose gel.

The prevalence of pathogens was calculated as infection rate (IR) and minimum infection rate (MIR). The IR and MIR for pooled ticks were determined by dividing the number of positive pools by the total number of pools of ticks tested or total number of tested ticks, respectively.

In order to analyze the association between human Lyme disease cases and *Borrelia* spp., the causative agents of Lyme Disease in ticks, statistical data on the occurrence of infectious diseases in Korea were obtained from the Infectious Disease Statistics, KDCA (https://npt.kdca.go.kr/pot/index.do, accessed on 1 September 2024). Monthly Lyme disease occurrence data from 2011 to 2024 were obtained, and the monthly average number of Lyme disease cases was calculated, and compared with the monthly positive rates of *Borrelia* spp. in ticks.

### 4.5. DNA Sequencing and Phylogenetic Analysis

The PCR products were purified using a LaboPass gel and PCR clean-up kit (Cosmogene Tech Co., Ltd., Seoul, Republic of Korea), and sequenced in both directions by Macrogen (Macrogen Inc., Seoul, Republic of Korea). The amplified sequence was identified using the basic local alignment search tool network service (https://blast.ncbi.nlm.nih.gov/Blast.cgi, accessed on 1 September 2024), and the identified sequences were aligned using BioEdit Sequence Alignment Editor software (version 7.2.5, Ibis Biosciences Co., Carlsbad, CA, USA). Phylogenetic analysis was performed using the maximum likelihood (ML) tree [42] and bootstrap of the MEGA X program. Genetic distance was calculated using MEGA X, and topologies were evaluated using bootstrap analysis with 1000 iterations.

### 4.6. Data and Statistical Analysis

Statistical data on the occurrence of infectious diseases in Korea were obtained from the Infectious Disease Statistics, KDCA (https://npt.kdca.go.kr/pot/index.do, accessed on 1 September 2024). Statistical analysis was performed with Pearson’s chi-square test using GraphPad Prism software (ver. 7.03). Statistical difference was considered significant at *p* values less than 0.05.

## Figures and Tables

**Table 1:** Number of ticks collected in Gangwon state, Korea.

Species	Developmental Stage	Heongseong	Yanggu	Hwacheon	Chuncheon	Total
*Haemaphysalis longicornis*	Adult (M)	21	38	6	67	132
	Adult (F)	152	134	72	640	998
	Nymph	2504	1402	516	7174	11,596
	Larva	236	836	123	673	1868
	Subtotal	2913	2410	717	8554	14,594
*Haemaphysalis flava*	Adult (M)	0	0	1	3	4
	Adult (F)	2	1	4	2	9
	Nymph	82	26	17	29	154
	Subtotal	84	27	22	34	167
*Ixodes nipponensis*	Adult (M)	0	3	7	4	14
	Adult (F)	0	0	3	4	7
	Nymph	0	0	1	1	2
	Larva	0	0	0	0	
	Subtotal	0	3	11	9	23
Total		2997	2440	750	8598	14,784

**Table 2:** Monthly number of larvae, nymph, and adult Ixodid ticks collected in Gangwon state, Korea.

Species	Developmental Stage	Mar	Apr	May	Jun	Jul	Aug	Sep	Oct	Total
*Haemaphysalis longicornis*	Adult (M)	0	19	36	46	31	0	0	0	132
	Adult (F)	0	119	163	366	309	40	1	0	998
	Nymph	0	1326	4037	4877	1032	167	145	12	11,596
	Larva	0	0	17	178	201	406	833	233	1868
	Subtotal	0	1464	4253	5467	1573	613	979	245	14,594
*Haemaphysalis flava*	Adult (M)	0	1	1	0	0	0	2	0	4
	Adult (F)	0	1	1	1	0	0	3	3	9
	Nymph	0	39	66	35	10	0	2	2	154
	Subtotal	0	41	68	36	10	0	7	5	167
*Ixodes nipponensis*	Adult (M)	5	2	2	0	0	0	1	4	14
	Adult (F)	1	0	0	0	0	0	2	4	7
	Nymph	1	1	0	0	0	0	0	0	2
	Subtotal	7	3	2	0	0	0	3	8	23
Total		7	1508	4323	5503	1583	613	989	258	14,784

**Table 3:** Infection rates of tick-borne pathogens in ticks collected in Gangwon state, Korea.

Species	Developmental Stage	No. of Tested Ticks	No. of Tested Tick Pools ^a^	*Theileria* spp.	*Borrelia* spp.	*Anaplasma* spp.
				No. of Positive Tick Pools (IR%, MIR%)		
*Haemaphysalis longicornis*	Adult (M)	71	28	2 (7.14, 2.82)	2 (7.14, 2.82)	0 (0.00, 0.00)
	Adult (F)	509	118	20 (16.95, 3.93)	2 (1.69, 0.39)	9 (7.63, 1.77)
	Nymph	5809	222	28 (12.61, 0.48)	11 (4.95, 0.19)	3 (1.35, 0.05)
	Larva	942	35	8 (22.86, 0.85)	1 (2.86, 0.11)	3 (8.57, 0.32)
	Subtotal	7331	403	58 (14.39, 0.79)	16 (3.97, 0.22)	15 (3.72, 0.20)
*Haemaphysalis flava*	Adult (M)	4	4	0 (0.00, 0.00)	0 (0.00, 0.00)	0 (0.00, 0.00)
	Adult (F)	8	8	1 (12.50, 12.50)	0 (0.00, 0.00)	0 (0.00, 0.00)
	Nymph	82	20	0 (0.00, 0.00)	1 (5.00, 1.22)	0 (0.00, 0.00)
	Subtotal	94	32	1 (3.13, 1.06)	1 (3.13, 1.06)	0 (0.00, 0.00)
*Ixodes nipponensis*	Adult (M)	13	13	1 (7.69, 7.69)	0 (0.00, 0.00)	0 (0.00, 0.00)
	Adult (F)	5	5	1 (20.00, 20.00)	0 (0.00, 0.00)	1 (20.00, 20.00)
	Nymph	2	2	0 (0.00, 0.00)	0 (0.00, 0.00)	0 (0.00, 0.00)
	Subtotal	20	20	2 (10.00, 10.00)	0 (0.00, 0.00)	1 (5.00, 5.00)
Total		7445	455	61 (13.41, 0.82)	17 (3.74, 0.23)	16 (3.52, 0.21)

^a^ Half of the collected ticks were pooled, each pool consisted of 1–5 adult ticks, 1–30 nymphs, or 1–50 larvae, and were used for the detection of pathogens, IR: infection rate, number of positive pool(s)/total number of the tested tick pools × 100%; MIR: minimum infection rate, number of positive pool(s)/total number of tested ticks × 100%.

**Table 4:** Primer information for the detection of tick-borne pathogens from the collected ticks in Korea.

Target Species	Primers	Sequences (5′-3′)	Target Gene	Size (bp)	Ref.
*Borrelia* spp.	Borrelia IGS-F	GGGTAATTAGTATTAGTCAGCTTA	*flagellin* B 5S-23S IGS	413	[38]
	Borrelia IGS-R	GCTTTAAGGCGAAGAAGGTCG			
	B5S-23S_F	CTGCGAGTTCGCGGGAGA		225–266	[39]
	B5S-23S-R	TCCTAGGCATTCACCATA			
*Anaplasma* spp.	EE1	TCCTGGCTCAGAACGAACGCTGGCGGC	16S rRNA	1433	[40]
	EE2	AGTCACTGACCCAACCTTAAATGGCTG			
	EE3	GTCGAACGGATTATTCTTTATAGCTTGC		926	
	EE4	CCCTTCCGTTAAGAAGGATCTAATCTCC			
*Babesia*/*Theileria* spp.	RLB-F2	GACACAGGGAGGTAGTGA CAAG	18S rRNA	460–540	[41]
	RLB-R2	CTAAGCATTTCACCTCTGACA GT			

## Data Availability

The data presented in this study are contained within the article.
